# Parasitiformes (ticks) and Acariformes (mites) vectors and their vertebrate host diversity: A global scoping review

**DOI:** 10.1016/j.onehlt.2025.101278

**Published:** 2025-11-21

**Authors:** Rashmi Bhat, Prakash Narayanan, Mohammed Mudassar Chanda, Michael Walsh

**Affiliations:** a Centre for One Health, Prasanna School of Public Health, Manipal Academy of Higher Education, Manipal, Karnataka, India; b Prasanna School of Public Health, Manipal Academy of Higher Education, Manipal, Karnataka, India; c ICAR-National Institute of Veterinary Epidemiology and Disease Informatics, Bengaluru, Karnataka, India; d The University of Sydney, Faculty of Medicine and Health, School of Public Health, New South Wales, Australia

**Keywords:** Biodiversity, Tick-borne diseases, Mite-borne diseases, Dilution effect, Species diversity, Public health

## Abstract

**Background:**

While the infection ecology and epidemiology of some vector-borne pathogens have received extensive research focus, many tick- and mite-borne zoonoses, particularly in the global South, have been largely neglected. The current scoping review aims to understand the global state of knowledge for the infection ecology and epidemiology of tick- and mite-borne pathogens, contextualised by their maintenance community composition, in the landscapes in which these pathogens circulate.

**Methods:**

Online databases were searched with keyword combinations to gather evidence on the relationship between vertebrate host species diversity and the occurrence of tick- and mite-borne diseases. Information related to pathogen, vector species involved in transmission, geographic location, and ecological and epidemiological relationships between host species diversity and tick- and mite-borne disease was recorded.

**Results:**

A total of 5510 papers were initially selected for screening based on this search with a final total of 36 papers included in the review. The review found that the literature is highly skewed toward Lyme disease (29 out of 36 studies) and focused on the global North regions (32 out of 36 studies). A general lack of studies on mite-borne diseases globally was evident. Additionally, 42 % of all studies reported evidence for the dilution effect whereby greater species richness in the maintenance community appeared to diminish pathogen transmission, while 31 % of the studies identified both dilution and amplification effects in the disease systems.

**Conclusions:**

While there has been an increase in studies on tick-borne diseases other than Lyme disease in the past decade, the current scoping review has identified an urgent need to study tick- and mite-borne diseases in the global South. The state of knowledge on these unique vector-pathogen systems with respect to relevant maintenance communities is sparse and requires targeted investigation in regions of the global South experiencing rapid habitat loss and subsequent changes in vertebrate species diversity.

## Graphical abstract

**Figure:**
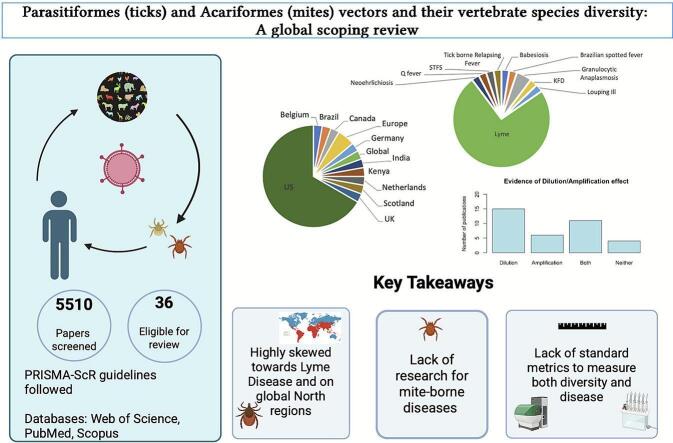

## 1 Introduction

Pathogens vectored by widespread ticks and mites cause some of the world's most burdensome diseases, making them medically, economically, and socially important [[1], [2], [3]]. The common names “ticks” and “mites” refer to the superorders Parasitiformes and Acariformes, respectively. These two superorders were previously considered as a monophyletic group under the subclass, Acari; however, more recent evidence indicates that this is a polyphyletic grouping [4,5] of these arachnids. Importantly, ticks and mites exhibit distinct evolutionary pathways, with varied morphological, biological, and ecological strategies despite species in both groups becoming major disease vectors. Hereafter, the Parasitiformes and Acariformes are referred to as “ticks” and “mites”, respectively, to avoid confusion with common usage. Species in both superorders are responsible for diseases of high public health importance, such as Crimean-Congo hemorrhagic fever (CCHF), scrub typhus, Lyme disease, and tick-borne encephalitis, which cause varying levels of morbidity and mortality [6]. For example, an estimated one million scrub typhus cases occur every year globally [7], while CCHF, although associated with substantially lower incidence, can have an average mortality rate of 10–40 %, but can reach as high as 60–80 % in some regions [8]. These diseases can be economically impactful as well with Lyme disease known to have an aggregate cost of $1 billion annually [9].

Anthropogenic driven changes like climate change, fragmented ecosystems, and shifts in land-use patterns can have significant public health implications. Temperature shifts can change the epidemiology and infection ecology of vector-borne diseases like malaria, leishmaniasis, filariasis as well as tick-borne haemorraghic fevers mainly by way of an increase in vectorial capacity and favorable habitats for vectors [10]. While there is a lack of longitudinal datasets that implicate increased tick- and mite-borne disease risk with climate change [11], vectors such as ticks and mites, being poikilothermic, are sensitive to changes in macro and micro-climate, as per their physiological requirements [12,13].

Increasing scientific evidence supports the association between diversity in ecological communities and the transmission of vector-borne pathogens [14]. Intra- and interspecific interactions within wildlife host communities can affect host-pathogen interactions and impact pathogen transmission dynamics in these communities. Over the past one and a half decades, evidence has linked species diversity loss in maintenance communities with increases in spillover and zoonosis incidence [15,16]. More specifically, the association may follow from a phenomenon known as the diversity dilution effect. However, evidence supports the converse in some pathogen systems, a phenomenon known as the diversity amplification effect [17,18].

The diversity dilution effect describes an inverse relationship between maintenance community species diversity and pathogen transmission (and a consequent reduction in spillover) via distinct mechanisms such as encounter reduction, host regulation, and vector regulation. Encounter reduction corresponds to an average decrease in encounters with infectious hosts among the members of an ecological community, resulting in reduced pathogen transmission. Host and vector regulation corresponds to trophic modulation of host and vector abundances through increased competition and predation, which may be more pronounced or varied in more species-rich communities [16,19]. Though these mechanisms are studied in detail in the Lyme disease system, there has been a recent increase in the literature investigating dilution in other pathogen systems [20,21]. Several meta-analyses and synthesis studies have found widespread evidence for the dilution effect in both animal and plant systems [[22], [23], [24], [25]] suggesting that this ecological phenomenon may be robust across multiple host-pathogen systems. Conversely, the diversity amplification effect describes a direct relationship between host diversity and pathogen transmission (and a consequent increase in spillover) [19]. The amplification effect is plausible when increased host species diversity would lead to higher parasite abundance or diversity through an increased presence of potential host species [26], which may lead to increases in novel pathogen exposures and increased spillover risk [27]. Evidence of host diversity positively affecting parasite diversity has been recorded in many studies [28,29] although such increases in pathogen richness can still decrease pathogen transmission for a given pathogen [30]. A global scale study [31] also found that regions with high levels of host species diversity were significantly correlated with zoonotic emerging infectious disease events. Nevertheless, the dilution and amplification effects are heavily context-dependent, whereby the extent to which diversity influences pathogen transmission in ecological communities and subsequent spillover depends on the particular infection systems and scale of investigation [17,32,33].

Owing to challenges in detecting and measuring both species diversity and infection, several metrics have been used as proxies to quantify both species diversity and infection occurrence. Consequently, differences in metrics can contribute to differences in the conclusions drawn with respect to the operation of dilution and amplification effects in host-pathogen systems [34,35]. Species diversity is usually measured as species richness and/or species evenness [36]. Species richness is the count of the number of species in a community, whereas evenness is the extent to which species abundances are evenly distributed in the community or dominated by one or a few species. In studies of diversity-disease relationships, metrics for measuring disease occurrence have been highly varied and may include vector pool infection prevalence [37], density of infected vectors [38,39], the incidence of human infections [40], and even disability-adjusted life years (DALYs) attributable to human infection [18]. Metrics like parasite prevalence might be more responsive to species diversity in the maintenance communities than metrics like human-centric DALYs or even loss of host fitness [34]. Moreover, disease can affect host community composition by selecting host species better suited to withstand infection via ecological and evolutionary processes like selection, drift, and population migration [41,42]. Host competence – the quality that determines the extent to which the host can successfully transmit the pathogen to another host or vector – might then drive variation in different types of hosts such as those that can dilute or amplify infection, or in their functional role as either dead-end hosts or bridge hosts [43,44]. Fundamentally, definitive determination of a species' status as a host depends on how infection is measured. Pathogen isolation or polymerase chain reaction testing can establish infection susceptibility, pathogen shedding, and host competence, whereas seroconversion can only establish pathogen exposure and cannot in isolation distinguish between competent and dead-end hosts.

The appropriate use of metrics is not trivial [35] and has consequences for inferences made regarding infection transmission and important contributing ecological factors. Competition and predation among different groups of hosts and non-hosts within maintenance communities can variably impact infection transmission processes. For example, Halsey et al. [45] found evidence for dilution when they interrogated the impact of species richness and host abundance on the density of both infected and non-infected vectors, whereas they found evidence of amplification when they interrogated the same metrics of species diversity against a different metric of pathogen presence, i.e. vector infection prevalence. The choice of diversity and disease metrics can strongly influence the study outcome and hence, needs to be considered with respect to the disease systems in question.

All ecological relationships, particularly the study of host-pathogen dynamics and community diversity, must be considered within the context of spatial scale since all interactions between organisms and between organisms and their environment vary according to the scale at which the interactions are observed [46,47]. For example, it is well established that species richness can change non-linearly with spatial scale. Moreover, emergent ecological relationships can vary with scale where, for example, biotic interactions might predominate in processes of community assembly at local scales, while abiotic factors might be more influential to the available species pool for community assembly at broader scales [48]. The importance of spatial scale also holds true for the study of diversity-disease relationships [49].

Tick- and mite-borne zoonoses may represent an important neglected disease group in the global South. Their relationship with host diversity could present a key knowledge gap that may be critical for mitigating spillover. Given the contrasting findings in the literature on the diversity-disease relationship for extensively investigated tick-borne pathogen systems, there is a need to understand the state of knowledge for these systems collectively and to note any knowledge gaps. This scoping review aims to describe the global state of the knowledge based on the published literature of tick- and mite-borne pathogens and the broader ecological communities in which they circulate to identify possible disparities in research effort and the implications of such disparities for preventing spillover. While specific ecological relationships such as host regulation, encounter reduction, and vector regulation are some of the mechanisms through which different forms of species diversity could operate, the current scoping review applies to all such applications of host diversity within the context of tick or mite-borne disease ecology.

## 2 Materials and methods

The scoping review was performed following the PRISMA Extension for Scoping Reviews (PRISMA-ScR) guidelines [50] (Supplementary Materials Table S1).

### 2.1 Search strategy

**Fig. 1:**
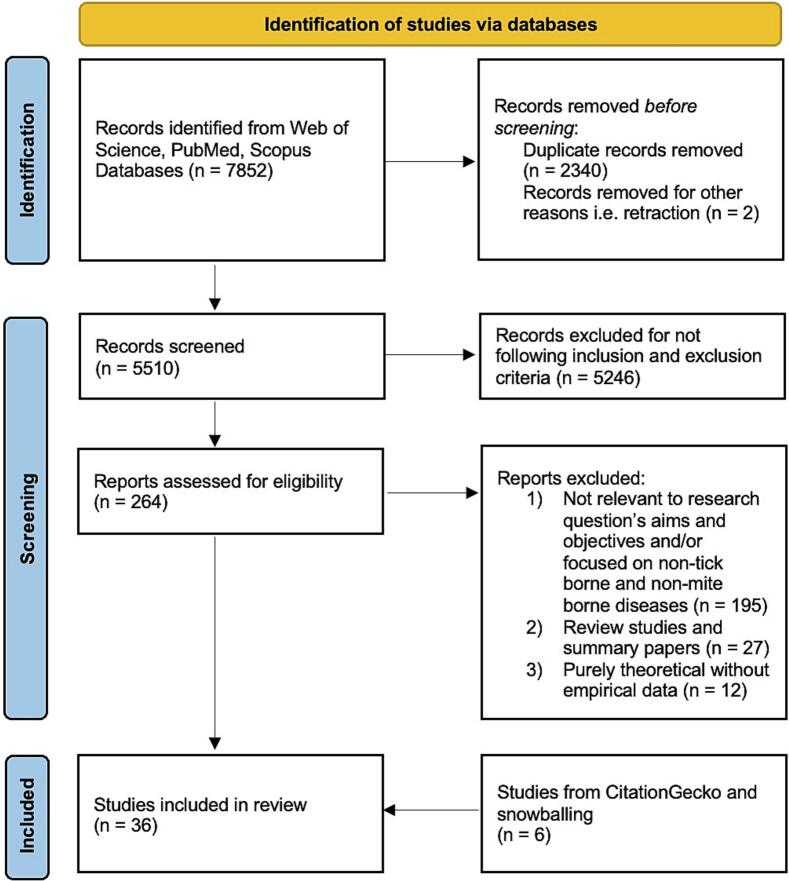
Preferred Reporting Items for Systematic Reviews and Meta-Analyses (PRISMA) flow diagram.

This study queried the Web of Science, PubMed, and Scopus databases through August 31st, 2023 to interrogate global disparities in tick- and mite-borne pathogen research and to investigate the relationship between these pathogens and species diversity in maintenance communities (Fig. 1). The search strategy included a combination of words representing the domains of vertebrate species diversity and tick- and mite-borne diseases. No restriction was placed on geographic location, year of publication or language during the search. The detailed search strategy for the databases is in Supplementary Materials Table S2.

### 2.2 Screening and data extraction

All database search results were imported into Zotero [51] to standardize citation formatting and remove duplicate entries. The consolidated citation list was then imported to Rayyan [52] for individual article screening by two independent reviewers based on title and abstract. While the databases index both English and non-English journals, all indexed records require English metadata including titles and abstracts. Articles published in other languages but with sufficient translation or summaries were screened for eligibility in the same manner as the English papers. Conference abstracts, grey literature, and opinion articles were excluded. Papers that used secondary data from other peer-reviewed literature were also considered. However, those studies that used only simulated data for modeling or were based purely on theory were excluded. Review studies such as meta-analyses and summary articles were also excluded. Studies were also excluded if they did not focus on tick- or mite-borne disease or if they were studies that synthesized research to understand the state of knowledge in the field or if they were only narrative studies. Those papers that measured disease occurrence solely as the presence of vectors were also excluded since presence or abundance of vectors need not always lead to disease occurrence.

The shortlisted papers were then screened for full-text eligibility. The resulting papers were then treated as “seed” papers in CitationGecko [53] and additional papers were added based on network analysis of the seed papers as well as snowballing. The literature citation analysis process in CitationGecko uses the relationship between papers that cite or are cited-by these seed papers. Highly cited papers are likely seminal while papers that are cited by the seed papers are perhaps ideas that are developing in the area. A network analysis of these connecting papers is useful in understanding the structure of the relationship between the papers and more importantly, any papers that might have been missed during the initial phase. The pathogen under study, the vector species primarily responsible for the pathogen transmission, the geographic location of the study, information regarding study scale, diversity and infection metrics used, and information related to dilution/amplification effects were extracted from each of the final papers and entered to a spreadsheet for analysis.

## 3 Results

A total of 7852 papers were considered after the database search – 2049 from PubMed, 4701 from Web of Science and 1102 from Scopus (Fig. 1). After removal of duplicates, 5510 results were imported to Rayyan for screening of title and abstract. A total of 264 papers were considered for full-text eligibility after which 234 papers were excluded. This resulted in 30 papers that were treated as seed papers for the network analysis in CitationGecko to identify potentially missed papers. The citation network analysis and snowballing recovered an additional six papers, yielding a final 36 papers for analysis.

### 3.1 Temporal trends

**Fig. 2:**
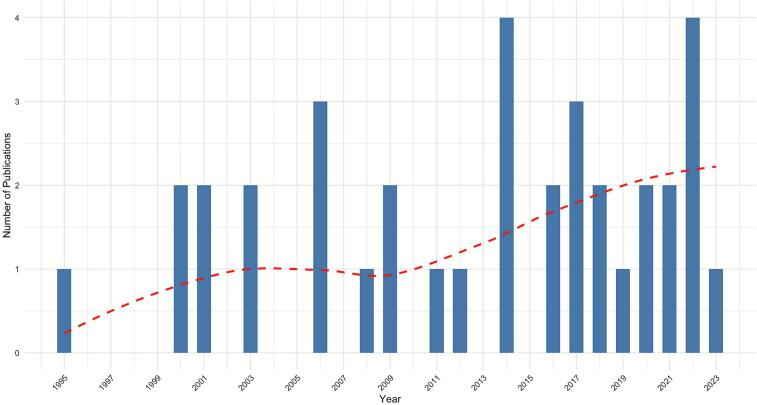
The number of publications investigating the relationship between occurrence of tick- and mite-borne disease with host diversity by year.

The earliest relevant paper described vertebrate species diversity and tick infection with the spirochete *Borrelia burgdorferi* in 1995 [54], and employed modeling of field data to conclude that the spirochete infection in ticks depended on the density and species composition of the vertebrate hosts. The literature on the subject increased after the publication of influential papers in 2003 [37] and 2006 [19] (Fig. 2). The latter paper discussed various mechanisms in which host diversity could increase or decrease pathogen transmission risk and laid the foundations for further exploration of the relationship between host species diversity and the prevalence of infection. A noticeable increase in publications occurred after 2019, suggesting renewed interest in ecological determinants of these vector-borne infections.

### 3.2 Taxonomic and thematic focus

Significantly, this scoping review found no studies focusing on host species diversity and mite-borne diseases. Though a recent study [55] found a positive relationship between rodent density and prevalence of *Orientia tsutsugamushi* infection in chiggers, there were no studies that have looked at the relationship between species diversity in the maintenance community and mite-borne diseases. The 36 studies included in this review all focused on tick-borne infections.

Among these studies, Lyme disease dominated representing 80.6 % (*n* = 29) of the studies, while only nine publications focused on diseases other than Lyme disease such as Babesiosis, Anaplasmosis, or Q fever (Table 1 and Supplementary Materials Table S3). From the years 1995 to 2010, 11 papers focused on Lyme disease but only two focused on other tick-borne diseases. The disparity has continued in more recent years from 2011 to 2023, with 18 papers focused on Lyme disease and seven focused on eight tick-borne diseases other than Lyme. The Gini coefficient (0.78) and coefficient of variation (225 %) signified strong inequality, which was confirmed by the chi-square test for uniformity (χ^2^(9) = 69.3, *P* < 0.001). However, five of the non-Lyme disease papers were published in the past four years, which may indicate gradual broadening of research interest beyond Lyme disease.

**Table 1:** Statistical summary describing the distribution of publications across infection types included in the scoping review.

Statistical Parameter/Test	Value
Total publications (N)	36
Mean publications/infection	3.6
Median	1
Mode	1
Range	28 (29–1)
Standard deviation (σ)	≈ 8.08
Coefficient of variation (CV)	≈ 225 %
Gini index (approx.)	≈ 0.78
Chi-square test for uniformity	χ^2^(9) = 69.3, *P* < 0.001

### 3.3 Geographic distribution of studies

**Fig. 3:**
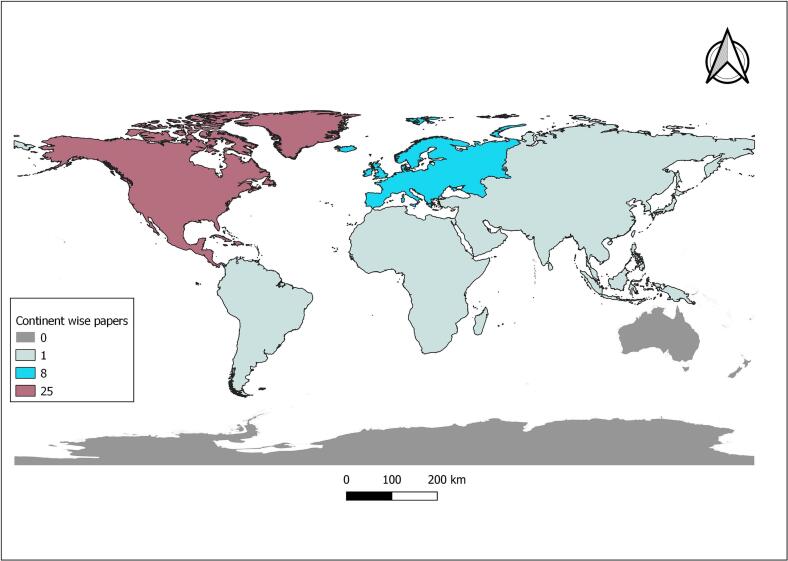
Continent-wise geographical representation of the studies included in the scoping review.

An overwhelming geographic disparity in research investigating the association between tick- and mite-borne disease/infection and host species diversity was apparent from this review. The results showed that 66.7 % (*n* = 24) of this work was based in the United States, and only three were based in the global South (Brazil, India, and Kenya) with 88.9 % of all studies based in the global North (Fig. 3).

### 3.4 Infection and diversity metrics

Various metrics were used to measure infection in the studies, chief among them was infection prevalence, which was recorded in 63.9 % (*n* = 23) of the papers. The other metrics were density of infected vectors (*n* = 10), number of human cases (*n* = 4), disease incidence in humans (*n* = 3), number of animal cases (n = 1), abundance of infected animals (n = 1), transmission probability (n = 1), and number of infectious hosts (n = 1). A χ^2^ goodness-of-fit test indicate a strong bias toward infection prevalence as the preferred measurement approach (χ^2^ (7) = 75.63, *P* < 0.001).

Detection methods showed similar concentration, Polymerase Chain Reaction (PCR) being the most frequently used (46.7 %, *n* = 14), followed by serology (*n* = 6); combined PCR and serology (*n* = 3). Some of the studies (*n* = 7) also used model-based estimates based on previously published data. Additionally, a few studies (n = 6), especially those that focused on the number of human cases, did not report how the underlying cases were tested. A χ^2^ goodness-of-fit test indicated a significant deviation from uniform use across methods (χ^2^ (4) = 14.2, *P* = 0.007), confirming a predominant reliance on molecular detection.

Infection was measured most often in vectors (only ticks since no studies on mites were found) (66.7 %, *n* = 24), followed by mammals (*n* = 11), humans (*n* = 7); and birds (n = 1), with no studies recording infection in reptiles, amphibians, or fish. The mean number of studies per host group was 8.6 (SD = 9.6), and a χ^2^ test confirmed a significant bias toward studies measuring infection in ticks (χ^2^ (3) = 24.8, *P* < 0.001).

Among the diversity metrics considered, species richness was the most common (80.6 %, *n* = 29) and was often combined with evenness (*n* = 5). The other metrics were Shannon diversity index (*n* = 8), abundance (*n* = 6), density (*n* = 4), Simpson's diversity index (n = 2), relative abundance (*n* = 1), host encounter abundance (n = 1). Only one study considered beta diversity and gamma diversity indices to measure species turnover and landscape level diversity, respectively. The mean number of studies per metric was 7.1 (SD = 8.6). A χ^2^ goodness-of-fit test confirmed significant unevenness in the application of diversity metrics (χ^2^ (7) = 41.2, *P* < 0.001), underscoring the disproportionate emphasis on species richness. Diversity was quantified primarily in mammals (80 %, *n* = 36), with fewer studies in birds (*n* = 13), lizards (n = 1), and vectors (n = 1). The mean number of studies per host group was 12.8 (SD = 15.6), and a χ^2^ test confirmed a significant bias toward mammalian hosts in diversity assessments (χ^2^(3) = 54.3, *P* < 0.001), highlighting a major taxonomic imbalance in current research.

### 3.5 Scale-specific analyses

The review also found only 13.8 % (*n* = 5) of the publications that considered the scale-specific nature of the diversity-disease relationship. Additionally, none of these papers explored more than two levels of spatial scales.

### 3.6 Evidence for dilution and amplification effects

The scoping review found 41.7 % (*n* = 15) of studies finding evidence for dilution effect, and 30.6 % (*n* = 11) of the studies finding evidence for both dilution and amplification effects in the disease systems (Table 2 and Supplementary Materials Table S4). Descriptive and inferential analyses indicate significant variation among categories (χ^2^ (3) = 9.33, *P* = 0.025). A binomial test further showed that studies reporting dilution effects were significantly more common than those reporting non-dilution outcomes (*P* = 0.042). These results together highlight the context dependency of the biodiversity-infection relationship across these disease systems, suggesting that both dilution and amplification effects can manifest depending on ecological and epidemiological conditions.

**Table 2:** Statistical summary of publication distribution across categories for evidence of dilution or amplification effects.

Statistical Parameter/Tests	Value
Mean publications per category	9
Median	8.5
Range	11 (15–4)
Standard deviation	≈ 4.30
Chi-square goodness-of-fit	χ^2^(3) = 9.33, *P* = 0.025
Binomial test (Dilution vs non-dilution)	*P* = 0.042

## 4 Discussion

The scoping review found several gaps in our current state of knowledge. Though there has been considerable debate on the relationship between species diversity in maintenance communities and pathogen transmission in those communities, much of what is known regarding this relationship comes from the Lyme disease system, which has been studied rigorously. While these Lyme disease studies have paved the way to understand the importance of ecological interactions involved in pathogen transmission in communities, there is a clear and critical need to diversify disease systems and geographic locations.

The disease system and geographic biases in diversity-disease relationship studies for tick- and mite-borne diseases has important implications. This already limited body of literature is firmly focused on global North regions and on the Lyme disease system, in particular. As such, there is a risk that results generated from this body of knowledge, though perhaps foundational for the field, may not adequately inform different pathogen systems in different parts of the world and may even misdirect future research efforts. Studies on applicability and transferability of research have often highlighted problems in the appropriate use of research results in local settings in other less developed countries [59]. Local conditions will undoubtedly have significant bearing on the diversity-disease relationships, and local priorities will determine how these relationships must be studied and understood. Moreover, neglect of vector-borne zoonoses in the global South tends to disproportionately affect marginalised people [60,61] who often live in close proximity to high biodiversity areas experiencing fragmentation that is due to regional and global economic forces that are outside of their control or influence. Finally, research inequity is exacerbated by a scarcity of resources, particularly the availability of a specialised labour force and the governmental or institutional funding necessary to conduct field- and laboratory-based surveillance [62]. Local researchers from the global South are key to diversifying the understanding of critical ecological relationships for infection transmission with implications for both public health and biodiversity conservation.

The review also highlighted substantial inconsistency in how both diversity and disease are quantified and reported. Lack of standardization impedes quantitative comparison across systems and limits the potential for synthesis. The metrics of pathogen presence and host species diversity must be chosen with a clear understanding of what these are representing and how they will impact on the inferences made based on the data generated. Studies have found that both species diversity and species abundance in maintenance communities can influence pathogen transmission and subsequent spillover risk for humans and their livestock [[63], [64], [65], [66], [67], [68]]. Additionally, most of the studies in the current review measured infection in vectors, mammals, or humans with only one study that measured infection in birds. Since there is no available data representing measured infection in other vertebrate groups like amphibians, fish and reptiles, there is a huge gap in our understanding on the diversity-disease relationship for these groups. Some vectors prefer certain vertebrate groups (eg: the tick *Amblyomma gervaisi* prefers snakes and *Varanus* species of lizards) and in the absence of research focusing on these groups, crucial data that can better inform diversity-disease relationship is missing. For example, understudied groups such as parasitic mites belonging to Histiostomatidae infest fish that can cause indirect consequences such as aquaculture contamination or potential allergic reactions in humans [69,70].

The mixed outcomes for evidence of dilution and amplification effects underscore the context-dependence of diversity-disease interactions. A single disease system could manifest both dilution and amplification effects. For example, [40] found a negative relationship between small mammalian species richness and the number of human Lyme disease cases per capita. Following the assumption that terrestrial small mammals, birds (ground-nesting, shrub-nesting, and ground-foraging), and lizards are the most important hosts available to the tick species in the Lyme disease system, they also found a positive relationship between species richness of ground dwelling birds and the number of human Lyme disease cases. This paper also found a negative relationship between species richness of lizards and human Lyme disease cases, which highlights the importance of evaluating variable impacts of different host groups within the maintenance community. Another study, through simulations, found that while species richness was associated with reduced density of infected vectors, species evenness was not [38]. Yet another study simulated wildlife host communities with varying densities of six small to medium sized mammalian hosts to measure the effect of species richness and abundance of these mammalian hosts on infection risk measured via density of nymphs, density of infected nymphs and nymphal infection prevalence [45]. The study found that greater species richness and host abundance were associated with reduced density of nymphs as well as density of infected nymphs, thus corresponding to a dilution effect. However, greater species richness and host abundance were also associated with increased nymphal infection prevalence, which corresponds to an amplification effect. More recently, Gonzalez-Bario et al. [71] found a quadratic relationship between the number of wild mammalian species and number of *Coxiella burnetii* infections reported in the mammalian species via serology and polymerase chain reaction. They found that *Coxiella burnetii* infections increased with mammalian species diversity up to an intermediate level of diversity but then decreased at high levels of mammalian diversity. Though not a quadratic relationship, in their global analysis, Miao et al. [72] found that in regions with <20 mammal species, mammalian species richness was positively related to the presence of severe fever with thrombocytopenia syndrome while it was negatively related in regions with ≥ 20 mammal species. These findings suggest that both community composition and host functional traits mediate the net effect of diversity, cautioning against over-generalisation of the dilution hypothesis.

Landscape structure can also modulate the relationship between species richness and disease risk. For example, Kyasanur Forest disease virus (KFDV) outbreaks have been shown to be associated with mammalian species richness, but the direction of the association was dependent on forest loss. One study found that increased species richness was associated with increased KFDV outbreak risk, but this positive association only applied to landscapes with minimal forest loss [73]. In landscapes with fragmented forest, lower species richness was associated with increased risk. Alternatively, ecological restoration efforts might also have important consequences on disease risk. Native habitat rehabilitation, especially in urban and sub-urban landscapes, that aims to restore ecological integrity can provide short and long ranging mitigation effects on the diversity-disease relationship and as such a careful assessment of such restoration efforts is required [74]. For example, one study found that habitat restoration via removal of an invasive tree had the benefit of averted human Lyme Disease cases [75], while another found that removal of an invasive grass increased the mortality rate of ticks [76]. Restoration measures such as those aimed at increasing habitat connectivity might facilitate wildlife movement and dispersal creating changes in wildlife host communities [77]. Vectors and pathogens that depend on these hosts for their survival might then impact infection transmission hazards and risks [[78], [79], [80]]. However, the complex impact of habitat restoration on the interactions between vertebrate communities, vectors and pathogens is unclear and hence, integrated surveillance measures are required to avoid inadvertent increase in infection risks [74].

Though the importance of spatial scales in the diversity-disease relationship has been reiterated in many studies [23,49,81], the current review recorded only five studies that explored scale-specific questions for the diversity-disease relationship for tick- and mite- borne diseases. A study found that host species richness was negatively correlated with the number of Lyme disease cases at the scale of state but was positively correlated at the scale of county [82]. However, in a meta-analysis that investigated the effect of spatial scale and latitude on the diversity-disease relationship across disease systems found that the dilution effect operated at multiple spatial scales, albeit attenuated in sub-tropical and tropical zones as compared to temperate zones [23]. Future research must incorporate multi-scale analyses to clarify these interactions.

Surprisingly, the review found no relevant studies explicitly examining the relationship between host diversity and mite-borne diseases, despite their significant global burden [7]. This gap could partly be due to the small size of the mites compared to other medically important vectors like ticks and fleas, making it very difficult to collect and study them for morphological and molecular identification and characterization [83]. Scrub typhus, transmitted by Trombiculidae mites infected with *Orientia tsutsugamushi* exemplifies such challenges with small mite vectors [7]. Mite populations can maintain the infection transovarially without vertebrate hosts, contributing to persistent transmission cycles. Despite the high morbidity and mortality associated with the approximately one million annual cases [7], studies exploring the role of host species diversity in scrub typhus ecology are scarce. Limited scientific evidence suggests that small mammal host abundance can influence the presence of mite islands [84], yet the mechanisms linking host community structure to infection risk remain poorly understood. Some recent studies [55,85] have found a relationship between rodent density and mite-borne disease risk, suggesting the influence of host community composition on mite infection dynamics. While Trombiculidae do exhibit specific habitat preferences that in turn influence host presence and selection, they are also known to thrive in transitional zones resulting in interactions with other incidental hosts [[56], [57], [58]]. The nature of these interactions is unclear and merits further scientific research.

## 5 Limitations

While the current scoping review tried to be inclusive and comprehensive, the interdisciplinary nature of the question might have resulted in certain papers being missed. The body of work on the subject is nascent and fast developing. Though non-peer reviewed sources like theses, conference abstracts and grey literature were not considered as part of the study, it is possible that high quality research results might have been reported in these sources of evidence. Nevertheless, the synthesis provides a robust baseline for identifying research priority gaps and guiding future research.

## 6 Conclusion

Current understanding of the relationship between host biodiversity and tick- and mite-borne disease systems remains narrow and geographically skewed. The review underscores the connections between ecological diversity, vector-borne disease risk, and global health equity, but emphasises the challenges in adequately describing these connections in under-resourced areas. Advancing this research requires (i) expanding research beyond Lyme disease, (ii) enhancing geographic representation, (iii) multi-scale analyses, and (iv) standardising methodological approaches to quantify both infection and diversity metrics. These steps are crucial to building a globally inclusive, ecologically grounded One Health framework for vector-borne diseases.

The following are the supplementary data related to this article.

Supplementary material Supplementary Table S1. PRISMA-ScR checklist
Supplementary Table S2. Search strategy for the databases included in the scoping review
Supplementary Table S3. The number of publications that studied the relationship between the infection and the vertebrate host diversity
Supplementary Table S4. The number of publications based on the evidence for dilution and/or amplification effects Supplementary material

## CRediT authorship contribution statement

**Rashmi Bhat:** Writing – review & editing, Writing – original draft, Validation, Methodology, Formal analysis, Data curation, Conceptualization. **Prakash Narayanan:** Writing – review & editing, Validation, Data curation. **Mohammed Mudassar Chanda:** Writing – review & editing, Validation, Formal analysis, Data curation. **Michael Walsh:** Writing – review & editing, Validation, Supervision, Methodology, Data curation, Conceptualization.

## Consent for publication

Not applicable.

## Ethics approval and consent to participate

Not applicable.

## Funding

RB and PN were supported by the DA Prasanna Endowment Research Grant. The funders had no role in study design, data collection and analysis, decision to publish, or preparation of the manuscript.

## Declaration of competing interest

The authors declare that they have no known competing financial interests or personal relationships that could have appeared to influence the work reported in this paper.

## Data Availability

The datasets generated and/or analysed during the current study are available in the figshare repository, https://doi.org/10.6084/m9.figshare.25249216
