# Supplementary material for: Parasitiformes (ticks) and Acariformes (mites) vectors and their vertebrate host diversity: A global scoping review
Source: One Health. 2025 Nov 21;21:101278. doi: 10.1016/j.onehlt.2025.101278 (PMC12701980; doi:10.1016/j.onehlt.2025.101278)
Supplement: Supplementary material — Supplementary Table S1. PRISMA-ScR checklist Supplementary Table S2. Search strategy for the databases included in the scoping review Supplementary Table S3. The number of publications that studied the relationship between the infection and the vertebrate host diversity Supplementary Table S4. The number of publications based on the evidence for dilution and/or amplification effects [file mmc1.docx]

**Supplementary materials**

**Table S1. Preferred Reporting Items for Systematic reviews and Meta-Analyses extension for Scoping Reviews (PRISMA-ScR) Checklist (adapted from Tricco et al. 2018)**

| **SECTION** | **ITEM** | **PRISMA-ScR CHECKLIST ITEM** | **REPORTED ON PAGE #** |
| --- | --- | --- | --- |
| **TITLE** | | | |
| Title | 1 | Identify the report as a scoping review. | See Title |
|  |  | “Arachnid-borne diseases and species diversity among vertebrate hosts: A scoping review of the global state of knowledge” |  |
| **ABSTRACT** | | | |
| Structured summary | 2 | Provide a structured summary that includes (as applicable): background, objectives, eligibility criteria, sources of evidence, charting methods, results, and conclusions that relate to the review questions and objectives. | See Abstract |
| **INTRODUCTION** | | | |
| Rationale | 3 | Describe the rationale for the review in the context of what is already known. Explain why the review questions/objectives lend themselves to a scoping review approach. | See Introduction |
| Objectives | 4 | Provide an explicit statement of the questions and objectives being addressed with reference to their key elements (e.g., population or participants, concepts, and context) or other relevant key elements used to conceptualize the review questions and/or objectives. | See Introduction |
| **METHODS** | | | |
| Protocol and registration | 5 | Indicate whether a review protocol exists; state if and where it can be accessed (e.g., a Web address); and if available, provide registration information, including the registration number. | N/A |
| Eligibility criteria | 6 | Specify characteristics of the sources of evidence used as eligibility criteria (e.g., years considered, language, and publication status), and provide a rationale. | See Methods, search strategy section |
|  |  | Database searches were performed on August 23^rd^, 2023. There was no restriction placed on geographic location, year of publication or language during the search |  |
| Information sources | 7 | Describe all information sources in the search (e.g., databases with dates of coverage and contact with authors to identify additional sources), as well as the date the most recent search was executed. | See Methods, Search Strategy section |
|  |  | PubMED, Web of Science, Scopus were searched. CitationGecko was used to identify any additional papers |  |
| Search | 8 | Present the full electronic search strategy for at least 1 database, including any limits used, such that it could be repeated. | Supplementary document 2 |
| Selection of sources of evidence | 9 | State the process for selecting sources of evidence (i.e., screening and eligibility) included in the scoping review. | See Methods, Screening and Data Extraction section |
| Data charting process | 10 | Describe the methods of charting data from the included sources of evidence (e.g., calibrated forms or forms that have been tested by the team before their use, and whether data charting was done independently or in duplicate) and any processes for obtaining and confirming data from investigators. | See Methods, Screening and Data Extraction,  Data Analysis section |
| Data items | 11 | List and define all variables for which data were sought and any assumptions and simplifications made. | See Methods, Data Analysis section |
|  |  | Data extracted on pathogen, vector species responsible for pathogen transmission, geographic location of study, details of diversity-disease relationship, scale specific details, metrics used |  |
| Critical appraisal of individual sources of evidence | 12 | If done, provide a rationale for conducting a critical appraisal of included sources of evidence; describe the methods used and how this information was used in any data synthesis (if appropriate). | N/A |
| Synthesis of results | 13 | Describe the methods of handling and summarizing the data that were charted. | See Methods, Data Analysis section |
| **RESULTS** | | | |
| Selection of sources of evidence | 14 | Give numbers of sources of evidence screened, assessed for eligibility, and included in the review, with reasons for exclusions at each stage, ideally using a flow diagram. | See Figure 1 |
| Characteristics of sources of evidence | 15 | For each source of evidence, present characteristics for which data were charted and provide the citations. | See Figure 1 |
|  |  | All online sources were merged for analysis. Duplicates were removed using Zotero |  |
| Critical appraisal within sources of evidence | 16 | If done, present data on critical appraisal of included sources of evidence (see item 12). | N/A |
| Results of individual sources of evidence | 17 | For each included source of evidence, present the relevant data that were charted that relate to the review questions and objectives. | See Figure 2 and Tables 1-3 |
| Synthesis of results | 18 | Summarize and/or present the charting results as they relate to the review questions and objectives. | See Figure 2 and Tables 1-3 |
| **DISCUSSION** | | | |
| Summary of evidence | 19 | Summarize the main results (including an overview of concepts, themes, and types of evidence available), link to the review questions and objectives, and consider the relevance to key groups. | See Discussion |
| Limitations | 20 | Discuss the limitations of the scoping review process. | See Discussion |
| Conclusions | 21 | Provide a general interpretation of the results with respect to the review questions and objectives, as well as potential implications and/or next steps. | See Discussion and Conclusion |
| **FUNDING** | | | |
| Funding | 22 | Describe sources of funding for the included sources of evidence, as well as sources of funding for the scoping review. Describe the role of the funders of the scoping review. | See Funding section |

Additional file 1: Table 1: PRISMA-ScR checklist

References:

Tricco AC, Lillie E, Zarin W, O’Brien KK, Colquhoun H, Levac D, Moher D, Peters MDJ, Horsley T, Weeks L, Hempel S, Akl EA, Chang C, McGowan J, Stewart L, Hartling L, Aldcroft A, Wilson MG, Garritty C, Lewin S, Godfrey CM, MacDonald MT, Langlois E V., Soares-Weiser K, Moriarty J, Clifford T, Tunçalp Ö, Straus SE (2018) PRISMA extension for scoping reviews (PRISMA-ScR): Checklist and explanation. Annals of Internal Medicine 169:467–473; https://doi.org/10.7326/M18-0850

**Table S2. Search strategy for the databases included in the scoping review**

|  | Concept | Search terms |
| --- | --- | --- |
| #1 | Vertebrates | vertebrate* OR mammal* OR bird* OR reptil* OR amphibian* OR fish* |
| #2 | Species diversity | “species diversity” OR diversity OR “species richness” OR richness OR “species evenness” OR evenness |
| #3 | Tick-borne diseases | “tick-borne disease*” OR “tick borne disease*” OR tick* |
| #4 | Mite-borne diseases | “mite-borne disease*” OR “mite borne disease*” OR mite* OR chigger* OR “chigger-borne disease*” OR “chigger borne disease*” |
| #5 | Arachnid-borne diseases | arachnid* OR “arachnid-borne disease*” OR “arachnid borne disease*” |
| #6 | General terms | “dilution effect” OR “amplification effect” OR “decoy effect” |

The final search string was #1 AND #2 AND (#3 OR #4 OR #5 OR #6).

Minor changes were made based on the database used. For example, MeSH terms were used for PubMed searches.

**Table S3.** The number of publications that studied the relationship between the infection and the vertebrate host diversity. Note that the sum of publications in the table is greater than the total publications included in this review since some studies have focused on multiple infections.

| **Infection** | **Number of publications** |
| --- | --- |
| Babesiosis | 1 |
| Brazilian Spotted Fever | 1 |
| Granulocytic Anaplasmosis | 2 |
| Kyasanur Forest Disease | 1 |
| Louping Ill | 1 |
| Lyme Disease | 29 |
| Neoehrlichiosis | 1 |
| Q fever | 1 |
| Severe Fever with Thrombocytopenia Syndrome | 1 |
| Tick borne Relapsing Fever | 1 |

**Table S4.** The number of publications based on the evidence for dilution and/or amplification effects

| **Evidence of dilution/amplification effect** | **Number of publications** |
| --- | --- |
| Dilution | 15 |
| Amplification | 6 |
| Both | 11 |
| Neither | 4 |
| **Total** | **36** |
